# Challenges on optimization of 3D-printed bone scaffolds

**DOI:** 10.1186/s12938-020-00810-2

**Published:** 2020-09-03

**Authors:** Marjan Bahraminasab

**Affiliations:** 1 Nervous System Stem Cells Research Center, Semnan University of Medical Sciences, Semnan, Iran; 2 Department of Tissue Engineering and Applied Cell Sciences, School of Medicine, Semnan University of Medical Sciences, Semnan, Iran

**Keywords:** Customized bone scaffold, Computational design, Composites, Functionally graded materials, Additive manufacturing, Bioprinting, Metadata analysis

## Abstract

Advances in biomaterials and the need for patient-specific bone scaffolds require modern manufacturing approaches in addition to a design strategy. Hybrid materials such as those with functionally graded properties are highly needed in tissue replacement and repair. However, their constituents, proportions, sizes, configurations and their connection to each other are a challenge to manufacturing. On the other hand, various bone defect sizes and sites require a cost-effective readily adaptive manufacturing technique to provide components (scaffolds) matching with the anatomical shape of the bone defect. Additive manufacturing or three-dimensional (3D) printing is capable of fabricating functional physical components with or without porosity by depositing the materials layer-by-layer using 3D computer models. Therefore, it facilitates the production of advanced bone scaffolds with the feasibility of making changes to the model. This review paper first discusses the development of a computer-aided-design (CAD) approach for the manufacture of bone scaffolds, from the anatomical data acquisition to the final model. It also provides information on the optimization of scaffold’s internal architecture, advanced materials, and process parameters to achieve the best biomimetic performance. Furthermore, the review paper describes the advantages and limitations of 3D printing technologies applied to the production of bone tissue scaffolds.

## Background

Bones in human body are prone to damage due to different causes such as fractures, diseases, and infections. Nevertheless, they have a remarkable capacity to repair and heal themselves after trauma and illness. Large defects, however, are never completely reinstated because their sizes are beyond the limit up to which the bones can repair [1]. In these conditions, therefore, a medical remedy is required to stabilize, align and support the damaged bone region to restore the lost function. Bone autografts are considered the gold standard treatment. However, they have a number of shortcomings including the limited sources and donor site morbidity. Allografts also have the risk of immune rejection and disease transmission [2, 3]. Therefore, the research has headed for other solutions via tissue engineering. Bone tissue engineering provides three-dimensional (3D) structures called scaffolds for new bone tissue regeneration using biomaterials, cells, and growth factors. To achieve the optimal function, that is proper bone tissue repair, the material of a bone scaffold should possess favorable biological properties including biocompatibility, biodegradability, and osteoconductivity, and acceptable mechanical properties including strength, and stiffness/modulus of elasticity [4, 5]. Furthermore, from the structural point of view, a scaffold should have porous structure of appropriate interconnected pore networks and proper pore size for efficient mass-transport activities including nourishment of cells, exchange of nutrients and wastes, and cell migration [6, 7]. These requirements make the design process (material and geometry optimization) very complex and may prevent easy customization of a scaffold for a specific patient defect, particularly when using conventional manufacturing approaches. The conventional methods to fabricate scaffolds usually do not have sufficient control on scaffold architecture (chemical composition variations, amount of porosity, pore size, shape and their network) and lead to suboptimal 3D bone scaffolds. However, 3D printing or additive manufacturing technologies are relatively new approaches which are capable of fabricating customized scaffolds with precise control on structure and with advanced materials [8–12]. In these manufacturing processes, the physical objects are built layer-by-layer through the continuous addition of small amounts of material, based on programmed routine and a computer model. Medical image-based modeling is an effective tool that can be combined with 3D printing to generate a complex customized 3D scaffold, matching the defect shape in the anatomical structure [13]. Furthermore, the computer-aided-design (CAD) model derived from medical images can be efficiently used for systematic optimization of scaffold material and geometry which leads to minimized trial and error experimentation and reduced cost.

The present review paper provides information on the development of CAD models for the additive manufacture of bone scaffolds. This starts from the anatomical data acquisition to the final CAD model. It also discusses on how the internal architecture of scaffolds and materials should be optimized to achieve the best function. At the end, the paper describes the advantages and limitations of 3D printing technologies used for making bone scaffolds, and discusses on the optimization of their process parameters to achieve successful scaffolds.

## Computer modeling of customized bone scaffolds

The use of 3D printing technologies for medical applications is rather different from other engineering components, particularly for the devices which are intended to be used inside the human body. In this area, the objects (organs and tissues) already exist physically, thus the development of the tissue-replacing parts involves reverse engineering approach which begins with the anatomical data acquisition. The acquired data, however, requires extensive efforts before 3D printing to provide a format which is compatible with a CAD program. Customized or patient-specific scaffold geometry can be gained by applying CAD software along with known individual patient anatomy parameters related to the defect site. Computer modeling before 3D printing of a bone scaffold essentially have two distinct steps: (1) data acquisition, and (2) image processing and model generation. These are important steps because there is significant variation in bone anatomy between different patients, and various defect shapes and sizes exist [14, 15]. Therefore, care must be taken to do these two steps accurately as they affect the quality of the final medical model and product.

### Anatomical data acquisition

The anatomical data of the damaged bone can be commonly obtained using computed tomography (CT) or magnetic resonance imaging (MRI) technologies [16, 17]. The former is a good choice for hard tissues imaging and provides reasonably high-resolution images. In this imaging method, differentiation of tissue is carried out through contrast segmentation and the grayscale value of each voxel is identified by tissue density [18]. Therefore, CT is much more efficient in the modeling of sharply distinct density variations, for example the interface between bone and soft tissues. The latter approach (MRI), however, is preferable when soft tissues are involved because MRI is highly capable of differentiating the soft tissue types and distinguishing the boundaries of the tissues with similar density [19, 20]. Hence, for modeling the damaged bone and the replacing bone scaffolds, CT scans can be favorably used. CT images are obtained based on the absorption detection of an ionizing radiation (X-rays). The damaged bone is exposed to the radiation and CT scans are conducted to provide a series of two-dimensional (2D) images identifying a density map of the bone (in DICOM format). By stacking the acquired images, a 3D representation of the bone scanned area is gained.

### 3D reconstruction and CAD model

**Fig. 1:**
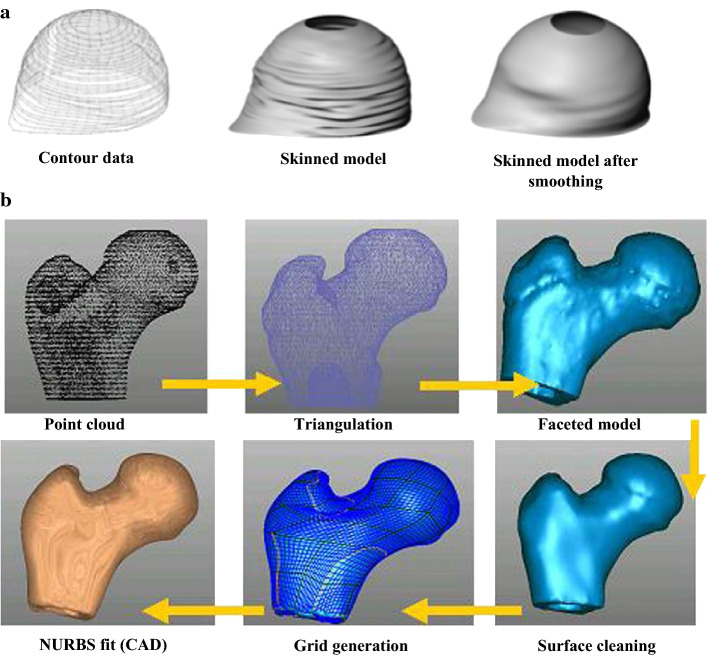
**a** 3D reconstruction of CT data (reprinted with permission from Springer Nature, J. H. Ryu et al. [23] copyright (2004)), and **b** reverse engineering approach to provide CAD model (reprinted from Sun et al. [19] Copyright (2005), with permission from Elsevier)

The 3D anatomical representation is usually built through either segmentation or volumetric representation [21, 22]. 2D segmentation is to extract the geometry of the object of interest, that is a bone region, from the CT scan data. The boundaries must be defined for each slice independently either by manual tracing or by edge detection using image-processing algorithms [23]. When a set of closed contours is gained, they are stacked in 3D and used as reference to form a solid model. This is usually carried out by skinning operations. After that, smoothing can be done to eliminate the wiggles from the skinned surface. Finally, the surface model is generated (Fig. 1a, adapted from [23]). 3D segmentation [24] of the CT data can recognize voxels bounding the bone and extract a tiled surface from them. Tiled surface is a discrete representation that typically consists of connected triangles. Once the segmentation and visualization are accomplished, the data can be translated into instructions for making of physical parts by 3D printing. Segmentation and 3D reconstruction can be done by MIMIC software. Volume representation involves volume rendering providing surfaces and the voxel-based representation [25, 26]. Volumetric imaging offers a 3D display with a continuum of image and surface intensity of data, however without explicitly defining a geometric surface in computer.

Design, analysis and optimization, however, require to be conducted in a computer-aided-design system and by providing CAD-based solid models which can be subsequently used in finite element analysis (FEA) software such as ABAQUS. There are several ways to generate CAD models from medical images including MedCAD interface approach, reverse engineering interface method and STL-triangulated model converting technique [19]. Among these methodologies, the reverse engineering approach is explained here, because the CAD models obtained by this method are much more stable in configuration, and there is less error in data transfer formats, particularly for an integrated CAD and FEA application. However, this process is relatively time-consuming despite the considerably better results. In the reverse engineering approach, a 3D voxel model generated from the segmentation is converted to point data, which is imported into a reverse engineering software such as Geomagic or Solidworks. These point clouds are then triangulated to create a faceted model. The freeform surfaces of non-uniform rational B-spline (NURBS) patches are used to fit across the outer shape of the model. Figure 1b shows this procedure which is adapted from [19].

By doing the above-mentioned steps, both the target bone with defect, and the isolated defect can be modeled. The whole model can be used later for analysis via FEA such as comparison of bone biomechanics before and after using scaffold and comparison with healthy bone, and the isolated defect model can be used to match the external shape of the designed scaffold. To design a successful bone scaffold, it is necessary to have knowledge on scaffold material and structural requirements. Therefore, the following section provides information on these requirements.

## Requirements for bone scaffolds and the clinical relevance

Clinical translation of bone scaffolds still deals with many challenges despite the intense efforts, and advancement over the past years. To develop more clinically practicable scaffolds, further investigations on right design and material properties are required which should be tailored in the scaffolds according to the different types of bone defects and fracture sites. Primarily, a material should be biocompatible and interact with living cells and tissues without provoking undesirable physiological responses. Biocompatibility encompasses all aspects dealing with the function of a biomaterial in human body [27]. This is a screening criterion which could be examined from different aspects (from in vitro cytotoxicity assay to in vivo acute and sub-chronic systemic toxicity) based on ISO 10993. Furthermore, it is desired that a bone scaffold material could be biodegradable, meaning that it can break down over time into non-toxic products capable of being metabolized and cleared from the body [28, 29]. Concurrently, the new tissue grows and gradually fills the defect. Other important factors are osteoconductivity, osteoinductivity, and osseointegration ability [30–33]. An osteoconductive material guides bone growth on its surface by supporting the growth of capillaries and cells from the host. Osteoinductive materials induce the osteogenesis process, by stimulating immature and pluripotent stem cells from a non-osseous environment to differentiate into chondrocytes and osteoblasts. These materials, therefore, allow regeneration in a place which normally does not heal if left untreated. Osseointegration occurs when direct functional and structural anchorage forms between an implant and bone. Lack of osseointegration, which is seen in bioinert materials, leads to the formation of a non-adherent fibrous capsule around the biomaterial a few weeks after implantation. Clinical complications of poor osseointegration, most often are due to mechanical instability. At the bone defect ends, where the bone interfaces the scaffold, there should be no micromotion which otherwise causes non-unions [34]. Furthermore, mechanical instability and micromotion may produce wear debris upon friction at the bone–scaffold interface and consequently result in lack of osseointegration and other adverse biological reactions. From the mechanical properties, strength and modulus of elasticity/stiffness of a bone scaffold material are of particular importance [5, 35]. Adequate mechanical strength provides integrity after implantation. Bone scaffolds are required to have temporary mechanical stability and withstand early biomechanical forces, such as wound contraction forces and body loads, as they degrade over time. Sometimes, in clinical practice a temporary external fixation is used to stabilize the bone. The mechanical strength depends on the material used and the manufacturing approach. For example, Peters et al. [36] used two different techniques including a conventional shaping technique (milling) and inkjet 3D printing (IJP) approach for building custom-made porous scaffolds from β-tricalcium phosphate (β-TCP). The authors showed that the 3D-printed scaffolds had much lower compressive strength, which was in the range of trabecular compressive strength (1.5–38 MPa [37]), but lower than that of cortical bone (100–150 MPa [38]). This restricts the use of these scaffolds at highly loaded site and suggests a post-treatment to enhance the mechanical properties. For bone tissue repair, the stiffness of bone scaffold should not be very low to provide mechanical stability, and should not be very high to cause stress-shielding. The elastic moduli of human cancellous and cortical bone tissues lie in the ranges of 10–1570 MPa and 14.9–35.3 GPa, respectively [37, 39]. Appropriate stiffness, close to that of bone at the defect site, is required to allow natural remodeling of the bone. This depends on the type of material and the porosity in the scaffold structure. Titanium and its alloys in the bulk/dense form have much higher elastic moduli (> 100 GPa) than the human bone. Therefore, despite their acceptable biocompatibility, stress-shielding phenomenon occurs which affects the bone remodeling. Numerous studies investigated on different porous Ti–6Al–4 V scaffolds using selective laser melting (SLM) technique (a 3D-printing method) and could successfully obtain low elastic moduli comparable to that of the cortical or the cancellous bone [39]. However, one challenge is to obtain high compressive strength because most of porous Ti–6Al–4 V structures have ultimate compressive strength values moderately lower than that of cortical bone. Other mechanical properties such as fatigue and creep resistances also seem to be essential over the long term [40, 41]. Scaffolds are subjected to cyclic loads during daily activity and they may experience fatigue failure [42]. At body temperatures, metals and ceramics are relatively resistant to creep [43] because it becomes significant at about 40–50% of their melting point [34], which is much higher than the body temperature. However, the creep behavior comes to be important generally above − 200 °C for polymers, thus it probably happens for these materials at physiological temperatures [34, 44, 45]. Mass transport is another issue that affects the nourishment of cells, exchange of nutrients and wastes, and cell migration [46, 47]. This can be addressed by structural design of bone scaffolds. Several factors related to porosity including pore size, pore shape, pore interconnectivity and amount of porosity can influence the success of a designed bone scaffold [48, 49]. In addition to mass transport, porosity has influence on different material properties of scaffolds such as stiffness and strength. The effect of porosity on the scaffold properties leads to a trade-off between the desired properties as the higher porosity, for example is favorable for mass transport while is unfavorable for mechanical function. One key point is the manufacturing route to make scaffolds because the exterior geometry of the defect is required to replicate. Readily adaptable processing helps in easy customization of different outer shapes of the defects and achieving a diversity of internal configurations [4]. Conventional methods, for instance those involving the molding technique, require the redesign and building of a new mold for each different geometry. Therefore, 3D printing technologies can efficiently be used to address this important aspect and to reduce cost. Furthermore, one can use 3D printing to control porosity and internal architecture of scaffolds.

It should be noted that fulfilling all these requirements may not be achieved by selecting a single uniform material or by any designed porosity provided by a conventional manufacturing approach. To achieve a bone scaffold with thorough function, there is a need for optimal porous structure, and optimal material ingredients provided by a precise advanced manufacturing approach, that is 3D printing. This has encouraged material engineers to utilize computer modeling and analysis for optimization, to develop hybrid porous materials such as composites [50], and hierarchical/functionally graded porous bone scaffolds [47], and to combine these with 3D printing technologies.

## Optimization for biomimetic function

### Internal structure

**Fig. 2:**
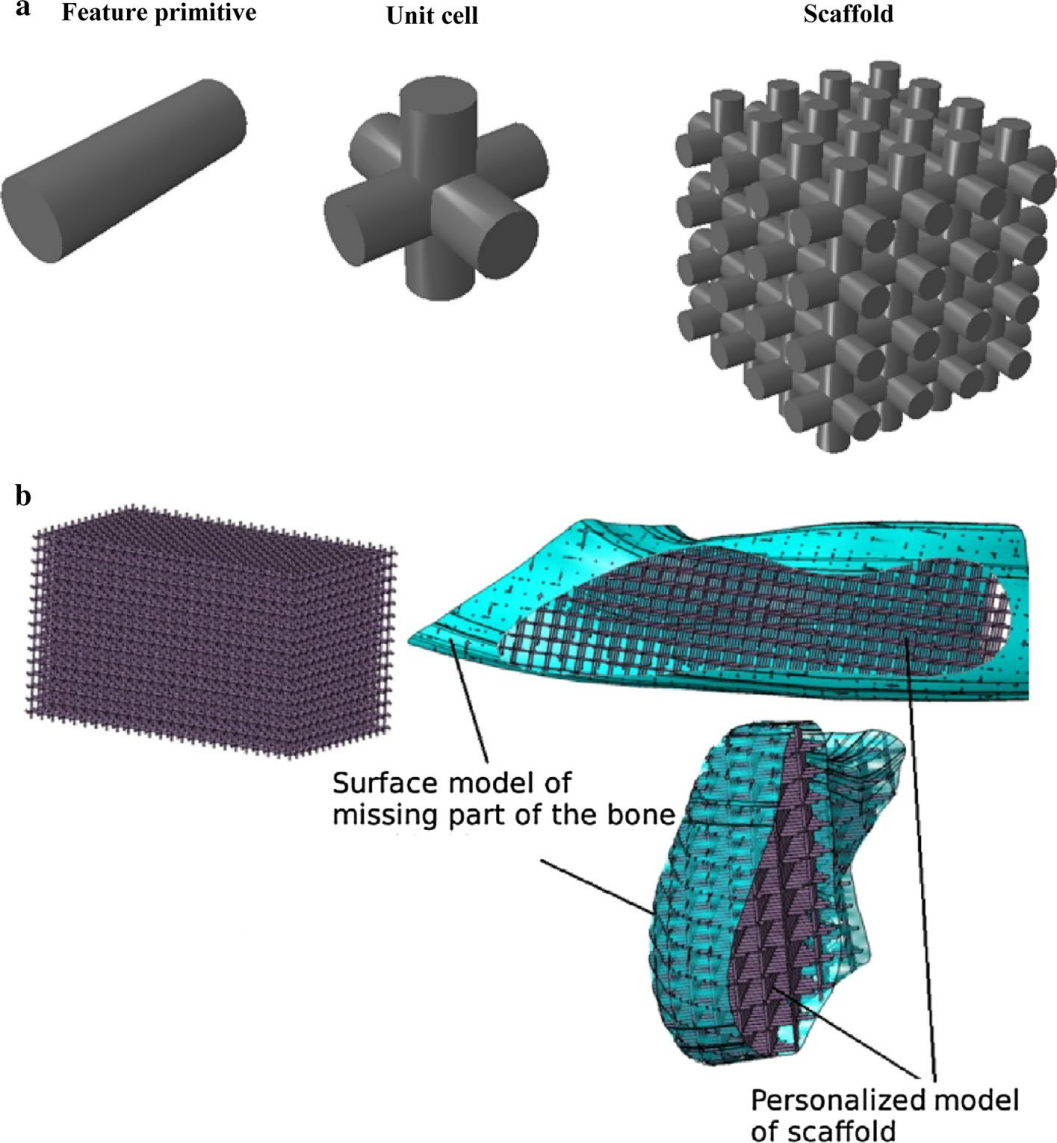
**a** The scaffold designed by periodic repeating of unit cells created in ABAQUS software, and **b** Boolean operation between the scaffold block model and the actual model of the mandible bone defect (reprinted from N. Vitković et al. [68] copyright (2018), with permission from Elsevier)

The internal architecture design of bone scaffold is a prime factor in successful function as it influences the mechanical characteristics of the scaffold and cell responses [47, 51–53]. To design the internal architecture, one can use unit cells as building blocks and then assemble them to form a 3D scaffold. These unit cells usually have hollow multi-dimensional/polyhedral shapes (Fig. 2a) and comprise feature primitives such as cylinder, for which the designers are capable of selectively changing their porous structure such as pore size and strut thickness. This enables them to adjust and tailor the properties of 3D scaffolds from different aspects including mechanical, physical, and biological. Therefore, it is possible to develop uniform and hierarchical structures. The natural tissues in human body such as bone usually have a gradient porosity, which causes the tissue to have gradient mechanical strength and stiffness. The radial and linear changes are observed in long, short and irregular bones, respectively. Therefore, the porous bone scaffold with gradient structure similar to that of the repairing bone is required [54]. The radial structural gradient can be achieved, for example by arranging struts of different thickness/diameter. Computer modeling of bone scaffolds based on FEA has been conducted in previous studies to evaluate the effect of different geometrical design (design variables) mostly on mechanical properties (objective functions) including compressive strength, stiffness, and effective bulk or elastic modulus [55–67].

From the biological point of view, appropriate transport of the nutrient and oxygen into the scaffold structure, and cell seeding are important in bone healing process. Fluid flow analysis, therefore, is an essential element in the internal design of bone scaffold. This can be done by combining CAD, FEA and computational fluid dynamics (CFD) [69, 70] to gain mechanical and micro-circulation properties including the stress–strain of the blood against the scaffold channels, and the blood flow velocity. The fluid flow characteristics depends on the scaffold pore size as the larger pores with higher permeability [71, 72] cause the cell suspension to move effortlessly through the scaffold structure. This may lead to higher average fluid velocities giving less time to the cells for adhesion to the scaffold surface [73]. It has been indicated that pore size and shape can affect the bone ingrowth [74, 75] as these influence the fluid flow characteristics, which may provide a shear stress possibly stimulating osteogenesis [76]. Another important biological aspect is that when a biodegradable bone scaffold is implanted in a given defect, both hydrolysis and bone remodeling processes start to happen. These also can be modeled, analyzed and optimized by FEA [77–79].

**Fig. 3:**
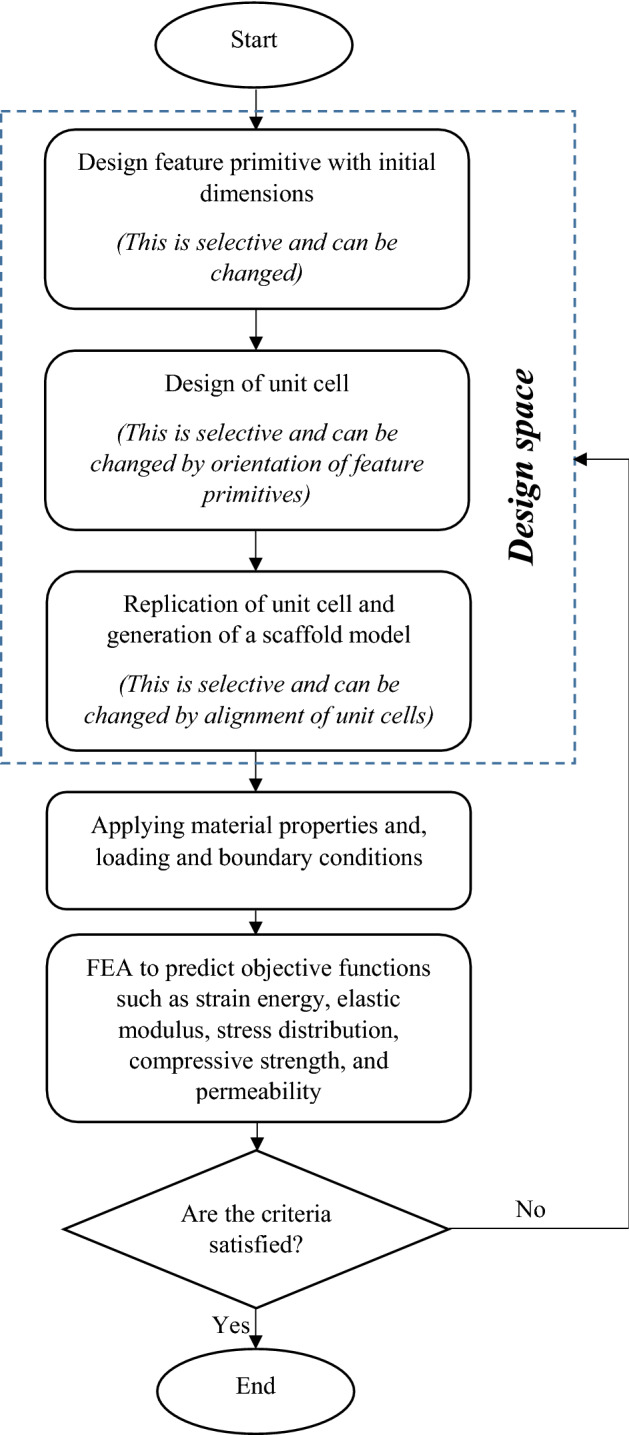
Optimization process of scaffold internal architecture

Despite the large number of investigations on different unit cells, the literature lacks optimization of geometrical parameters in bone scaffold considering fully mechano-biological criteria. The identification of the optimal value of all the scaffold geometrical features requires a systematic approach taking into account multiple responses with respect to different types of pore shapes and sizes. Several studies are now available in the literature using parametric analysis [80–86]. For example, one investigation by Boccaccio and the colleagues [80] developed an optimization algorithm which perturbs iteratively the unit cell geometry until the bone formation was maximized. The best way to optimize the bone scaffold geometry appears to be parametric finite element analysis considering all the variables (pore shape, pore size, porosity percentages, and pore interconnectivity) and multiple responses (mechanical properties, permeability and cell responses) together to achieve a thorough design. It is better for optimization to be done based on the given application to define appropriate loads and boundary conditions and tailor the best internal structure. However, it should be noted that multi-objective optimization with several independent variables is a challenging task which requires knowledge on advanced computer modeling and mathematical approaches. One way is to apply Design of Experiments (DOE) [7, 87–89] which is a systematic strategy that can proficiently determine all effects of design variables including main, interaction and quadratic, and in the meantime reduces the computational efforts while it is able to obtain the required information. Several software packages including Minitab and Expert Design are used for these purposes [90–92]. DOE has been used in the optimization process of scaffolds, however, mostly for finding optimal hybrid materials or process parameters [93–95]. The suggested optimization process of scaffold internal architecture is shown in Fig. 3.

Once the optimized internal architecture is obtained, it should subsequently match the external defect shape in the target anatomic structure, precisely. This can be done using Boolean operation between the scaffold block model and the actual model of the tissue defect which is isolated when providing CAD model (Fig. 2b, adapted from [68]).

One point that should be noted is that periodic distribution of unit cells with regular geometry are very sophisticated and allow easier modeling, optimization, and manufacturing. Irregular porous internal architecture has been also modeled to attain more similar structure to that of the replacing tissue, however by applying particular equations such as reaction–diffusion model [96]. Irregular porous internal architecture could be modeled for biomimetic printing of cortical and trabecular bones. It can also be useful when detailed biological features are involved in the model such as lacuna for osteocytes. Nevertheless, creation of the detailed model with irregularity and biological features is a real challenge and requires very sophisticated computational approaches.

### Advanced materials

There are a large number of materials including metals, ceramics and polymers used in bone scaffold tissue engineering. However, the existing single-constituent biomaterials fail in satisfying all the requirements and are unable to completely replicate the bone properties when used alone [97]. Therefore, they cannot optimally match the host bone. This is the motivation for developing new hybrid biomaterials including composites. Composite materials, which consist of two or more constituents, can be tailored to have spatial arrangement in order to provide desired properties in a single material system. Judicious selection of the constituents, and optimizing their volume fraction and orientation, during the design process of bone scaffolds can possibly lead to biomimetic design, for example it may provide the material degradation rate coinciding the target tissue regeneration [98]. Different types of composites including polymer–ceramic, polymer–metal, ceramic–metal, and polymer–polymer have been developed to achieve improved function. Polymer and ceramic are usually combined to make a compromise between the insufficient rigidity of the polymer caused mechanical instability, and the brittleness of the ceramic caused fracture. However, other characteristics such as hydrophobicity, low cell adhesion site, and little biological interactions of some polymers like polycaprolactone (PCL), also, can be modified by adding a ceramic like nano-sized hydroxyapatite (HA) [99]. Polymers and biodegradable metals including poly-L-lactic acid (PLLA) and magnesium (Mg), can also make composites to provide desired biodegradability rate and at the same time higher strength and structural integrity [100]. Furthermore, composites of ceramic and biodegradable metal such as magnesium–calcium phosphate can provide superior biocompatibility, biodegradability and faster and more efficient osteogenesis in vivo [101]. Polymer–polymer composites have also been extensively developed for making bone scaffolds with better function, examples are scaffolds made of polyethylene glycol (PEG)-PLA [102], PLA-polyaniline (PANI) [103], chitosan–collagen–hyaluronic acid [104] and many others. It should be pointed out despite the advantages offered by uniform composite, there is a trade-off between the material properties. One advanced generation of composites are functionally graded materials (FGMs) which have been investigated for bone tissue repair [105–107]. FGMs are non-uniform composites usually designed to have a chemical composition gradient and/or porosity gradient selectively in one or more direction, based on the required properties, in one material system [9, 108]. Such structure is usually seen in natural biological materials, and therefore is highly demanded. FGMs can be particularly useful for replacing the defect sites where a transition from bone to cartilage exists [109–113] such as osteochondral defects. The FGM for these defects should possess a hard material for bone replacement at one site and a flexible material for cartilage replacement at the other site.

**Fig. 4:**
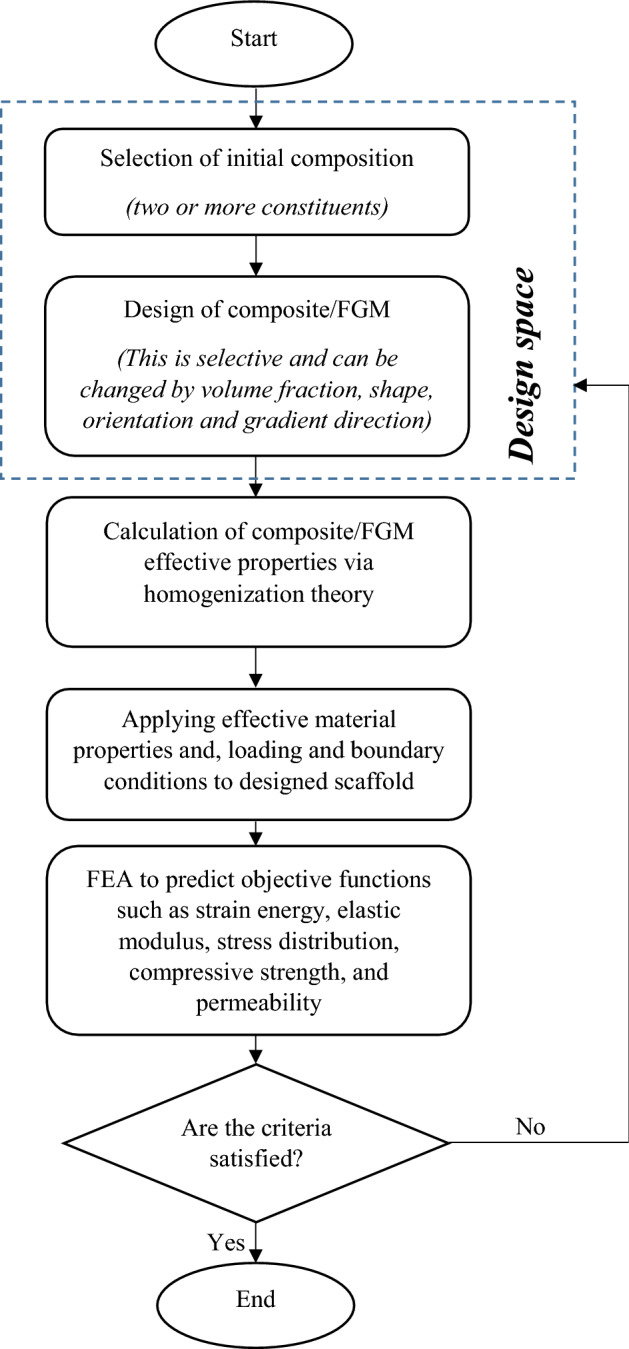
Optimization process of scaffold material

In the design stage of composites and FGMs, there are several variables that influence the final properties. To achieve the optimal material design, therefore, extensive laboratory efforts are required which involves too much cost. It seems rational to take the advantage of computer modeling and analysis to gain optimum solution and after that start the manufacturing process. Applying scaffold CAD model and FEA software aid in finding the best chemical composition, volume fraction, orientation and gradient direction considering multiple responses such as mechanical strength and bone remodeling. Figure 4 shows the general steps in optimization of hybrid materials (composites/FGMs) for scaffolds. From the manufacturing point of view, it is rather difficult to fabricate composites and particularly FGMs by 3D printing technologies. To provide bone scaffolds with functionally graded property through these contemporary approaches, there is a need for a CAD software that can define material gradient in the 3D solid model of a scaffold, and a 3D printing machine with multiple feeders/nozzles and mixing chambers [111, 114]. Zhang and Bandyopadhyay [115] utilized laser engineered net shaping (LENS), which is a laser-based 3D printing method, to fabricate a Ti–Al_2_O_3_ FGM with 4 layers. This material primarily was developed for load-bearing applications in orthopedics such as articular surfaces where the ceramic surface with high hardness provides high wear resistance at articulation and Ti surface interacts with the bone tissue [116, 117].

## 3D printing of bone scaffold based on CAD model

**Fig. 5:**
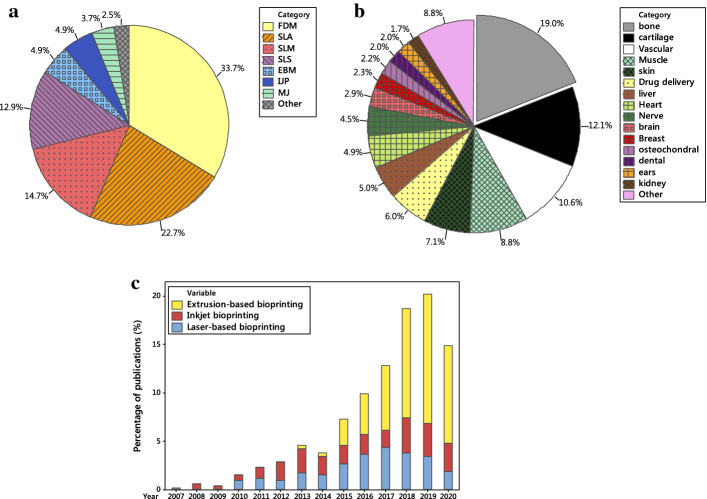
**a** Percentages of different 3D printing approaches investigated for bone scaffolds; **b** percentages of 3D bioprinting uses in different tissue engineering applications, and **c** comparison of uses of different 3D bioprinting approaches over time (based on Scopus search, type of document was article, keywords for **a** 3D printing and bone scaffold and the technique name, **b** 3D bioprinting with each application name, and **c** 3D bioprinting and name of approach)

3D printing techniques are modern manufacturing approaches which are programmed based on the generated computer models to fabricate 3D physical objects precisely fulfilling the individual customer (patient) needs. The 3D CAD models are used to manufacture the complex bone tissue scaffolds with the external shape matching the defect, and internal optimal porosity. Usually, the 3D bone scaffold model is converted to a number of triangular facets connected at the vertices, that is surface tessellation (STL) files, which is subsequently sliced horizontally using a computer algorithm. The sliced data are applied to provide information for layer-by-layer building of the final bone scaffold exactly replicating its 3D model. The 3D printing technologies for making bone tissue scaffolds are increasingly used and include a wide range of approaches (Fig. 5a). These methods, however, operate under the same principles. They use different types of materials including metals, polymers, ceramics, or even cells which are encapsulated within a bio-ink. The 3D printing approaches are classified from different perspectives. In the following subsections, two classifications based on stimulation used for integrating matter, and based on inclusion of cells are presented. Subsequently, the process optimization associated with these technologies is discussed.

### 3D printing technologies based on stimulation used

The 3D printing machines, based on the stimulation used for integrating matter, can be categorized into (1) laser-based 3D printing technologies which operate using laser stimulation to bond either material powders or fluid medium; (2) extrusion-based 3D printing technologies which extrude molten materials that either cool and physically bond or are further solidified by UV stimulation, and (3) ink-based 3D printing technologies which print liquid or aerosol chemical binders to chemically bond the material powders together. Laser-based technologies include stereolithography (SLA) [118–130], selective laser sintering (SLS) [131–136], electron beam melting (EBM) [137–139], LENS [140], SLM [39, 141–149], and two-photon polymerization (2PP) [150, 151]. Extrusion-based technologies include fused deposition modeling (FDM) [152], and material jetting (MJ) [96]. Ink-based technologies include IJP [153], and aerosol jet printing (AJP) [154]. Comprehensive descriptions of these technologies can be found in [2, 155–157], to name a few. However, the operation mechanisms of these techniques and their advantages and limitations are briefly described here.

#### Laser-based technologies

##### Stereolithography

**Fig. 6:**
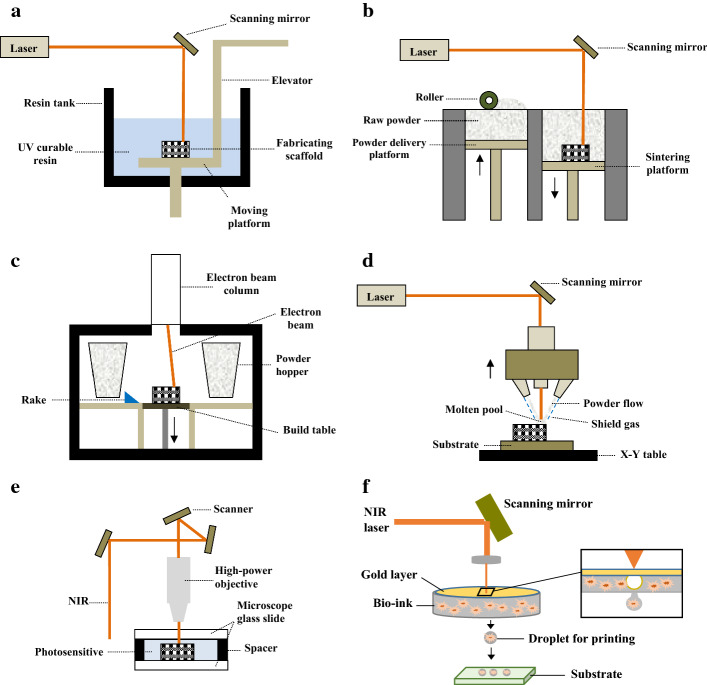
Schematic of laser-based 3D printing technologies: **a** SLA, **b** SLS/SLM, **c** EBM, **d** LENS, **e** 2PP, and **f** laser-based bioprinting

This technique makes physical objects by photo-polymerization of photosensitive resin using a laser beam (UV light). The photosensitive material is converted to solid on a platform bed when it is exposed to the laser and by movement of the laser beam based on the model first slice, a layer is formed. To build the successive layer, the platform is moved down and fresh liquid resin flows over the first layer which is then solidified and adheres to the previous layer. This is done iteratively to build the entire physical object matching the 3D CAD model (Fig. 6a). The SLA is able to fabricate complex internal features and large parts. Furthermore, the accuracy and resolution of SLA-built parts are high. The other advantage is that it can be adapted to be used for bioprinting. One limitation of SLA technique is the need for support structures to avoid the collapse under hydrostatic pressure. Usually, the support structures are difficult to be removed from the printed bodies. Other disadvantages include extensive cleaning procedures, chemical reactions with ambient air, restricted height of the printed part to the resin bath size, and resin waste [158].

##### Selective laser sintering

Selective laser sintering is very similar to SLA printing technique, however, the material used is in the form of powder (mostly polymer) which is first placed on a platform bed. Then, to build the model cross-sectional shape, a laser selectively sinters the powder by elevating the temperature to melt the powder surface leading to diffusion. The print platform is subsequently adjusted by descending and a roller spreads new powder layer over the prior working surface. The process is repeated until the stacked layers provide the final shape (Fig. 6b). The printed objects by SLS possess good mechanical properties and do not often need post-processing. For fabricating SLS parts, the support structures are not required and the technique is economic. However, the issues related to material wastage and difficulty in removal of the entrapped powder exist.

##### Electron beam melting

In the electron beam melting machine, as the name implies, accelerated electrons generated by an electron gun via heating a tungsten filament are used for integrating matter (metals). The electron beam scans the metal powders in a vacuum chamber to sinter them based on the given CAD model. The raw powders are gravitationally poured down from cassettes (powder hoppers) and raked to distribute onto the sintering platform for making the first layer or over the prior solidified layer for making the successive cross-section. The build platform moves down after building of each layer of the prescribed component, consecutively (Fig. 6c). The vacuum leads to reduced risk of reactive metals oxidation (such as titanium), decreased contamination and impurity-free objects. Furthermore, the printed objects have good mechanical properties. However, there is a need for support structures to avoid warping. Meanwhile, EBM is slow and expensive.

##### Laser-engineered net shaping

This technique is very similar to EBM, however there are some differences including use of a laser beam rather than an electron beam, motion control system, and material feeding approach. LENS uses metal or ceramic powders which are distributed on a table numerically controlled in XY plane. A laser beam is then directed on the powder to provide cross-sections of the model. First the material melts and a small molten pool is provided. After that, a certain amount of powders is fed directly into the molten pool as the table moves in X and Y directions according to the computer model, thus the volume increases. The molten material line is solidified rapidly when the laser beam moves away. After building a layer, the laser-head and the powder feeding nozzle move upward and provide a distance by the thickness of the next layer to generate it. This is repeated until the entire 3D object is produced (Fig. 6d). Good mechanical properties are shown by the objects printed by LENS. This technique can be used to fabricate composites and FGMs. The challenges in fabricating objects by LESN include the need for post-processing, cutting of the built part from the build substrate, and low-dimensional accuracy.

##### Selective laser melting

SLM machine, which is also known as direct metal laser sintering (DMLS), operates similar to SLS, however, it uses a laser beam for melting metal powder instead of polymer powder, on a build platform to print the 3D objects. SLM applies a high-power density laser that can melt metallic powders and fuse them together. Similar to EBM, the building of objects in SLM is conducted in a chamber of highly controlled atmosphere of inert gas. Nevertheless, its powder feeding system is similar to SLS having two platforms; one for dispensing powder and the other for building the component. In fact, a layer of metal powder is uniformly distributed onto a metal substrate plate fastened to a table with vertical movement. Each layer is selectively melted while the laser beam scans in X and Y directions. Figure 6a, which shows the SLS process, also can be representative of SLM because their difference is in the materials used. SLM-fabricated components exhibit high mechanical properties. In this technique, however, there is probability of warping and inconsistent mechanical properties due to non-uniform heat distribution. Similar to EBM, SLM is a slow and expensive 3D printing approach.

##### Two-photon polymerization

The 2PP technique provides the opportunity of fabricating components at a greater depth, higher resolution up to nano-level, and a fast speed for making small parts. In 2PP, a near-infrared (NIR) ultra-short-pulsed laser is concentrated into a volume of photosensitive solution to cause a phase change from liquid to solid by a polymerization process to fabricate 3D structures. 2PP is based on the concurrent absorption of two photons inducing chemical reactions. A high-precision stage (piezoelectric/PZT or linear motor-driven) is employed for movement of the fabricating sample across a fixed laser excitation beam (Fig. 6e). In fact, the table enables positioning in all directions. Single-photon absorption, which is used for example in stereolithography, is basically 2D, because the absorption of UV light by the resin occurs within the first few micrometers. However, the photo-curable resins are transparent in NIR region, thus the laser pulses are able to focus into the volume of the resin [159].

The 2PP system is able to print at the 100-nm level and is capable of integrating nano- and microscale features in the fabricating parts. The 2PP is possibly suitable for patterning tissue engineering structures with surface features [160, 161] in order to promote tissue repair. The controlled 3D topology provided by 2PP can positively influence cell–biomaterial interactions [162, 163]. Considering the typical size of bone scaffolds, this approach could be time-consuming and may not be cost-effective. For example, the time needed to make a 1-mm^3^ volume structure for use in microfluidics exceeds 104 days [164]. However, it could be possibly used along with other 3D printing methods for making bone scaffolds. Another limitation of 2PP technique is that multi-material printing is difficult.

#### Extrusion-based technologies

##### Fused deposition modeling

**Fig. 7:**
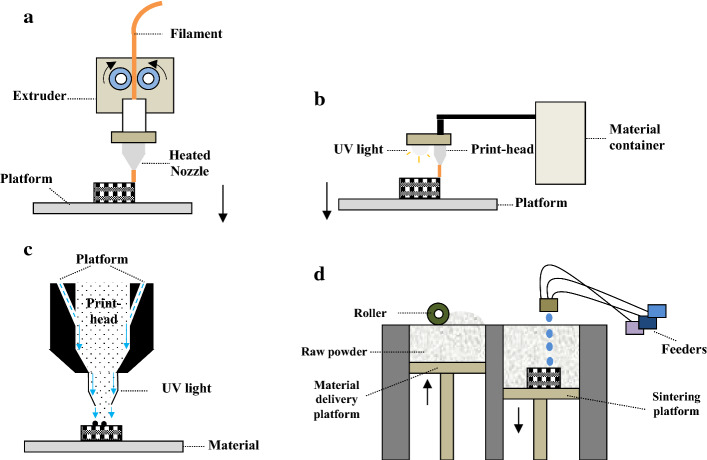
Schematic of **a** FDM, **b** MJ, **c** AJP, and **b** IJP

FDM process is used to manufacture objects like 3D bone scaffolds by melting an extruding material, which is typically a thermoplastic polymer, through a moveable nozzle onto a build platform. FDM uses a filament of material which goes through two rotating rollers and reaches into the extruder head, where it is melted. The nozzle movement is in X and Y directions making the filament to be deposited on the platform and build a parallel series of material deposits to finally provide the first layer of the component. Afterward, the platform moves down along the Z axis for manufacture of the subsequent new layer over the previous layer. When the deposited material cools, it solidifies and adheres to the prior layer. This is done iteratively until the final 3D structure is gained. Some models of FDM machines have two nozzles, one of which is used for depositing the filament material while the other is employed to extrude a temporary support material. The structures provided by support material are broken away or dissolved by a solution. Figure 7a shows schematic of FDM 3D printing system. The 3D structures fabricated by FDM usually have good mechanical properties such as those made up of PLA and PCL [165, 166]. Furthermore, the control over porosity and properties can be achieved by adjusting the printing speed. FDM is a well-known 3D printing technique which is easy to use, safe and reliable with a low purchase price. The FDM-printed objects can be handled almost directly after fabrication as the post-processing is not often required. However, sometimes a support is used and required to be removed or improved surface finish is desired. FDM machines have minimal material wastage because only the required amount of filament is used. A wide range of thermoplastic polymers such as PLA, PCL, and recently their composites with ceramics like HA [167] can be made by this printer in quite complex structure and superior chemical and physical functionality. This technique also has been used with other fabrication methods such as electrospinning [168] to provide more efficient bone scaffolds. FDM, however, is not suitable for printing most proteins and cells because heating is needed for providing the molten phase. Despite this, the technology could be adapted for bioprinting.

##### Material jetting

Material jetting technique extrudes liquid photopolymer through a print-head onto a substrate platform. This machine uses UV light to solidify the layers when they build up. The resin is first heated to attain ideal viscosity for printing, then the print-head moves over the platform to jet/deposit a number of photo-cure polymer droplets to the desired sites. A UV light source is attached to the print-head that cures and solidifies the deposited droplets of material to build the first layer. When the layer is completed, the platform moves down by a layer thickness and the process is repeated until the entire part is built. Figure 7b schematically shows MJ 3D printing system. MJ as an extrusion-based 3D printing technique is able to fabricate 3D scaffolds with flexible geometrical control. The manufacture resolution is in the range of tens to hundreds of microns which can meet the size demand for bone scaffolds. However, the objects printed by MJ usually exhibit poor mechanical properties. Furthermore, similar to FDM, it is limited in precisely mimicking the natural biological features in micron or submicron scale, if needed.

#### Ink-based technologies

##### Aerosol jet printing

Aerosol jet printing is a 3D printing technology which is able to print objects with small featured size in the range of 10 µm. Aerosol jet printing uses aerodynamic focusing to dispense a printing ink onto a substrate which can be either planner or non-planner as it has the ability to print at varying distances. The process begins with atomization process which causes the liquid ink to atomize into aerosol with a droplet size of 1–5 µm, using pneumonic or ultrasonic atomizers. The provided aerosol is then headed toward a print-head where it is aerodynamically focused by a sheath gas stream. The gas flow does not allow the aerosol to have contact with the nozzle print-head inner cladding. Figure 7c shows aerosol jet printing technique schematically. The objects obtained by AJP has higher resolution, and greater range of materials with significantly lower viscosities can be used than IJP [169]. This approach due to the atomization process might not be suitable for inclusion of biomolecule in the printing structures. Similar to 2PP approach, AJP could be time and cost consuming for making bone scaffolds where the required sizes are usually large. However, it can be used for patterning thin layer deposition [170]. The AJP printing also has low materials waste.

##### Inkjet printing

Inkjet printing is a drop-by-drop printing technique which can possibly create cellularized structures. Inkjet 3D printing, similar to MJ distributes droplets of ink, however to integrate powders. This process is similar to aerosol jet printing, but with no atomization process and with lower resolution. The print-head is located over a bed of powder and dispenses the ink onto powder through programmed routine to provide a particular shape related to the first cross-section (polymerization stabilizes the printed material in place). The print-head and the platform distance is then increased incrementally to roll a new powder layer over the stage for scanning the next cross-section which adheres to the previously printed layer. This process is repeated until the whole physical object is achieved. Figure 7d represents inkjet printing schematically. Inkjet 3D printing can have more than one print-head from which cells and biomaterials can be individually deposited under computer control. Therefore, IJP can be utilized for bioprinting [171], and is fast and cheap. However, it should be pointed out that the constructs built by IJP are often fragile and require post-processing to be strengthened. Table 1 represents some information about the advantages and limitations of all above-mentioned 3D printing approaches, and the materials used for printing objects.

**Table 1:** Summarized description of 3D printing technologies

Technology category	Technology name	Compatible materials	Advantages	Limitations
Laser-based	SLA	Liquid photopolymers	Obtaining complex internal features Ability to build large parts Bioprinting Good accuracy and high resolution	Need for support structures, not to collapse under hydrostatic pressure Difficulty in removal of support structures
	SLS	Polymer powders Ceramic powders	No need for post-processing No need for support structures Good mechanical properties Economic	Material wastage Difficulty in removal of the entrapped powder manually
	EBM	Metal powders	Good mechanical properties	Slow and expensive Need for support structures to reduce stresses and avoid warping
	LENS	Metal powders Ceramic powders	Good mechanical properties Ability to fabricate composites and FGMs	Post-processing is required Cutting of built part from the build substrate Low dimensional accuracy
	SLM		Good mechanical properties	Probability of warping and inconsistent mechanical properties due to non-uniform heat distribution Slow and expensive
	2PP	Photopolymer or hydrogel solutions	Good resolution enabling integration of nano-sized and microscale features	For bone scaffolding, should be used along with other 3D printing methods to provide favorable material properties
Extrusion-based	FDM	Polymeric and polymer-based composite filaments	Good mechanical properties Moderate speed enabling the control over porosity and properties Adaptable for bioprinting	Not suitable for printing most proteins and cells because the heating needed for providing molten phase
	MJ	Liquid photopolymers	No need for post-curing	Poor mechanical properties
Ink-based	IJP	Mostly hydrogels, but other polymers and ceramics are also used such as PCL, HA, bioactive glasses and PLA Metal nanoparticles can be incorporated such as silver	Bioprinting Fast and cheap	Constructs built are often fragile Need for post-processing to strengthen the constructs
	AJP		Higher resolution than Inkjet Printing Greater range of materials with significantly lower viscosities than inkjet printing	Not suitable for bioprinting due to necessity to atomize the inks Expensive

### 3D printing technologies based on cell inclusion

The 3D printing technologies can also be divided into the approaches that make the scaffolds from the biomaterials solely with the cell seeding done as a post-processing task, and to the so-called bioprinting [172–174] technologies in which the living cells can be incorporated into the fabrication process. The former group does not deal with the difficulty of maintaining cell viability through the production process and can be implemented to develop porous structure with a wide range of materials, even with natural polymers which have poor 3D printability. Indirect 3D printing also can be done via these approaches [175]. Indirect 3D printing builds a negative mold used for pouring the natural polymers which is the positive for the reproduction of the original scaffold. Then, the polymer scaffold (such as collagen) are removed from the mold through a drying process [176, 177]. This facilitates using the advantages of the natural polymers including good biocompatibility and favorable micro-environment for cells. Indirect 3D printing technique has also been used for other polymeric materials (synthetic polymers) to have controllable porosity [178, 179]. Bioprinting (the latter group) is an additive fabrication approach with the potential of building or patterning viable organ-like or tissue structures in 3D [180]. It has attracted the researchers to investigate different cell-laden structures for regeneration of many tissues in the recent years (Fig. 5b). In these approaches, generally, bio-inks are used to make scaffolds in a layer-by-layer manner. Bio-inks are a mixture of one or more biomaterials with living cells [181]. Biomaterials for bioprinting require to be processed with no adverse effects on the suspended cells, and to be strong enough when printed to maintain their shapes. Hydrogels (water swollen polymers designed to control a number of cellular functions including adhesion, spreading, proliferation and differentiation) are typically used as bio-inks for printing cells, morphogens, and growth factors in different tissue regeneration areas including cartilage, bone, bile duct, nerve, heart, etc.[182–191]. Different formulations have been used for making the bio-ink hydrogels. For example, Bendtsen et al. [184] developed seven different hydrogel formulations to find the optimal composition to make bone tissue scaffolds. Three of the hydrogels consisted of alginate, Na_2_HPO_4_, and CaSO_4_, three others had additional HA, and one had additional HA and NaCl. Furthermore, mouse calvaria 3T3-E1 (MC3T3) cells were incorporated in the optimal hydrogel (2.5% alginate, 0.15% Na_2_HPO_4_, 0.20% CaSO_4_, 2.5% HA) and 3D printed. In another study, Wehrle et al. [187] examined 3D bioprinting of hydrogel consisting of fibrin, gelatin, hyaluronic acid, glycerol and HA with incorporation of mesenchymal stem cells (MSCs). The scaffold showed promising results in terms of mechanical stability, cell viability and calcification after bioprinting. Incorporation of HA resulted in suitable viscoelastic properties along with excellent biocompatibility. Furthermore, Daly et al. [192] examined several bio-inks and found the optimal one to engineer endochondral bone formation and the whole bone organ. For the former purpose, 3D-printed PCL scaffolds combined with a bio-ink laden by MSCs through infusion. The compressive modulus of the constructs increased about 350-fold. For the latter purpose, a model of human vertebrae was prepared to print PCL and MSCs-laden bio-ink filaments rather than infusing the MSCs-laden bio-ink into a pre-printed PCL network. In this approach, the bio-ink filaments co-deposited alongside the PCL filaments in a layer-by-layer manner to build a composite structure for vertebrae. The in vivo results showed the development of a vascularized bone tissue having trabecular-like endochondral bone with a marrow structure. Furthermore, in situ printing of mesenchymal stromal cells along with collagen and nano-HA was conducted to obtain favorable bone regeneration, in a calvaria defect model in mice [193]. The results of this study showed that different cellular arrangements affect the bone tissue formation.

A number of 3D bioprinting systems has been developed which falls in the same categories as 3D printing techniques [193–201]. Among these techniques, the extrusion-based bioprinting has become more popular (Fig. 5c) because hydrogel precursors having low‐shear viscosities can be used for printing and it is able to deposit high cell densities, similar to the target tissue structure [202]. Although bioprinting techniques have the same principles of the methods explained in previous “3D printing technologies based on stimulation used” section, they may have some differences due to the need for adaptation not to adversely influence the living cells. For example, in the laser-based printing approaches, the laser directly focused on the raw matter to make a solid object. However, in laser-based bioprinting (also known as laser-assisted bioprinting) a NIR pulsed laser beam concentrated on a transparent quartz glass slide/ribbon coated with a gold layer to absorb the beam energy creating a cavitation in a thin layer of bio-ink which is spread on the ribbon. This indirectly drives a micro-droplet, having cells, towards the substrate (Fig. 6f). This nozzle-free technique is able to position multiple cell types, preserve the activity of encapsulated cells, and provide high spatial resolutions lower than 20 µm [203, 204]. Laser-based bioprinting method can also facilitate the positioning of a singular cell per droplet [193, 205]. Nevertheless, it is an expensive bioprinting process requiring a highly complex setup with low stability and scalability [203]. Furthermore, the long-term influence of laser exposure on the printing cells is not fully known and requires more investigations for example on genotoxicity [206–208]. The inkjet bioprinting techniques are similar to IJP and MJ, but neither to bind powders nor to be photopolymerized. This bioprinting technique is capable of producing objects with a spatial resolution between 50 and 300 µm [203, 209]. However, the undesirable aggregation of cells within the hydrogel can induce variations in droplet formation and change the trajectory, thus it influences the printing quality [204, 210]. Furthermore, printing of the highly viscous materials to make 3D constructs is a real challenge in inkjet bioprinting technique, thus this method is mostly used for building up small scaffolds [211]. One advantage of this method is the use of multi-nozzle printhead which decreases the printing time and facilitates the production of larger-scale cellular constructs [212]. The principle of extrusion-based bioprinting (also known as direct ink writing) technique is also same as the MJ process. The only difference is the materials used, which is a cell-laden bio-ink in the bioprinting. The key advantages of this technique are the control over the deposition and distribution of cells within the bio-inks, the possibility of printing very high cellular densities and multiple materials, and a superb structural integrity because of continuous bio-ink deposition [213]. These make the application of this technology very relevant for manufacture of scaffolds, despite the lower resolution (about 200 µm) compared to the inkjet (50–300 µm) and laser-based (< 20 µm) bioprinting techniques [207]. One limitation of this technique is that the mechanical-based extrusion mechanisms generate large driving forces and pressure drops at the nozzle which can induce cell apoptosis and rupture cell membranes [214, 215]. However, the pneumatic-based extrusion systems are more appropriate for highly viscous bio-inks because they can maintain a filamentous structure after deposition [204].

A main advantage of bioprinting compared to other 3D printing methods is the controlled allocation of cells within the scaffold structure during the manufacturing process. This avoids the need for post-processing cell seeding and the problems of non-uniform distribution and poor attachment of the cells [1]. The bioprinting, however, has limitations regarding the use of biomaterials and the processing parameters such as printing speed. The presently available hydrogels cannot offer adequate mechanical properties comparable with those of bone, and they have restrictions in providing large size scaffold because of slow print speed which is essential for cell viability [216, 217]. The rheological properties of the bio-inks are important in making them suitably printable. The presence of additional constituents to provide composite material or drug may induce changes on the ink rheological behavior which consequently influences the microstructure and the mechanical properties of the final scaffolds [218]. It has been shown that bio-ink molecular weight and crosslinking ratio affect the mechanical properties and fate of cells inside the printed tissue constructs [185]. Therefore, careful process optimization is required in addition to material and geometrical optimization due to the involvement of cells and biomolecules in the manufacturing process [219]. For making composite bone scaffolds, however, combining of multiple 3D printing techniques can be a possible way forward [220] where the hybrid bone scaffolds can be processed with desired size, and adequate mechanical characteristics, while filled with precisely positioned cells.

### 3D printing process optimization

**Fig. 8:**
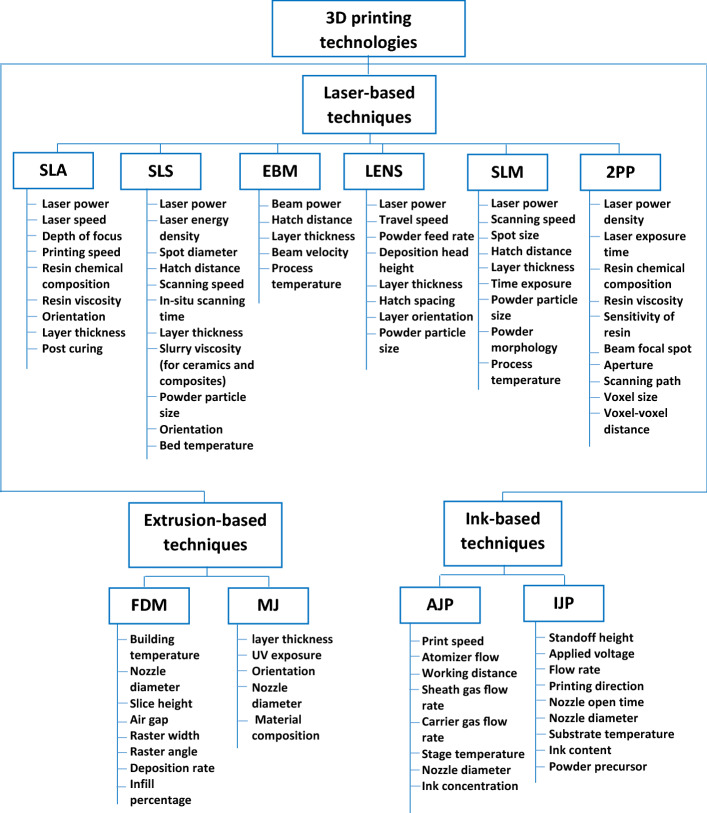
Process parameters and material variables for 3D printing technologies

In addition to materials and structural optimization, the process parameters should be adjusted and optimized to manufacture scaffolds with desired attributes. Numerous studies investigated the effect of process parameters on different responses such as mechanical properties (strength, elongation, Young’s modulus), surface roughness, resolution and dimensional accuracy, and printing quality in different techniques including SLA [221–224], SLS [225–234], EBM [235–241], LENS [242, 243], SLM [39, 244–250], 2PP [251–253], FDM [254–256], MJ [257, 258], AJP [259–262], and IJP [263–266]. There are many parameters in the 3D printing approaches which vary between different techniques as their machine structure, printing mechanisms, and materials used are rather different. Figure 8 summarizes the process and material variables for 3D printing technologies.

Although the factors in laser-based techniques are somewhat different, the laser variables are commonly considered. It has been reported that the most common process variables studied for Ti–6Al–4 V are laser power and scanning speed to find the optimal level of relative density, microstructure, surface roughness and mechanical properties [39]. In the printing approaches that use powder materials (SLS, EBM, LENS, SLM, and IJP), the characteristics of the powder such as particle size and morphology are important. One study on printing of β-tricalcium phosphate demonstrated that it is crucial for the printing process that the powder facilitates the formation of thin layers (100–200 μm thickness) with no grooves in the surface to achieve a favorable printing quality, which is associated with the powder particle size distribution (particle sizes between 20 and 50 μm with the absence of particles smaller than 5 μm) [267]. Another study also investigated the effect of SLM process parameters (layer thickness, laser power, point distance, exposure time, and hatching distance) for a range of particle size distributions of Ti–6Al–4 V and found that small powder particles helps in achieving higher density parts in a much shorter fabrication time [268]. Material viscosity is of importance in the printing methods such as SLA, 2PP, IJP and SLS where resins are used, or when ceramic or composite objects are intended to be made using slurries. One challenge in ceramic ink/slurry, such as zirconia and alumina slurry, is the rather low-volume fraction of solid constituent (ceramic loading) which is usually below 40 vol% [269]. This is particularly challenging for SLA as a result of the restricted solubility of the resin to ceramic powder. The solid loading of ceramic slurry could influence the accuracy of printed objects, the sintering shrinkage, and the mechanical behavior. One recent study by Brazete et al. investigated on the optimization of ZrO_2_ inks to build 3D porous load-bearing bone scaffolds [270]. They could successfully prepare ZrO_2_ inks with high solid volume fraction (48 vol%) which resulted in a satisfactory shape retention in the scaffolds with macropores of different sizes. Another challenge is that only relatively coarse particles can be used to gain a flowable slurry, which is essential for the layer deposition [271]. This hinders the densification during sintering and necessitates the use of additional post-treatment. It has been reported that the viscosity of ink/binder is also important in the dimensional accuracy of the printed β-TCP scaffolds by IJP; too low concentration resulted in irregular customized block forms due to irregular binder flow inside the powder pile, and too high concentration led to irregular and brittle scaffolds due to the blocking of inkjet printer [36]. Nozzle characteristics are also influential in 3D printing approaches including FDM, MJ, AJP, and IJP. For example, it has been reported that the FDM printing technique can accurately build scaffolds when the diameter of strands used to make PLA scaffolds were close to the nozzle diameter. On the contrary, in case of a large difference, fabrication errors were large and imposed on the filament diameter due to inappropriate material flow [272]. The strand diameters tested for nozzle diameter of 800 µm were 500, 600, 700, and 800 µm, and for nozzle diameter of 400 µm it was 400 µm. The strand diameters of 400 µm and ≥ 600 µm were well reproduced, but large reproduction errors were observed for the strand diameter of 500 µm. This happened because the material flow was only 39%, which was considerably smaller than 100%, thus the nozzle did not fill properly and consequently the filament could not form correctly. Furthermore, the nozzle diameter is very important in bioprinting where inappropriate diameter can induce mechanical damage to the cell membrane during 3D bioprinting process. Chang et al. [273] studied the effects of nozzle diameter (150, 250, 400 μm) and dispensing pressure (5, 10, 20, and 40 psi) of a 3D bioprinting technique on HepG2 liver cells recovery and proliferation. The results of their study showed that the mechanical damage to the cells due to increase in dispensing pressure or decrease in nozzle diameter, caused the cell viability to decrease (53.39% at 40 psi and 23.07% at 150 μm). Printing speed is another factor that affects the print quality and consequently the scaffold performance. In extrusion-based bioprinting process, the print speed and extrusion pressure interactively affect the strand diameter of the printing scaffold [274, 275]. It has been indicated that printing at lower extrusion pressure needs a slower print speed while printing at higher extrusion pressure requires a faster print speed to obtain mechanically stable structure. Furthermore, higher pressure reduces the cell viability percentage [274, 276]. Billiet et al. [276] investigated the 3D bioprinting of gelatin methacrylamide cell-laden constructs using a new photoinitiator (VA-086). The effect of several factors including hydrogel concentration, printing pressure, printing speed, printing temperature and cell density on construct architecture were analyzed. Finally, the scaffolds could be printed with a 100% pore interconnectivity in the gelatin concentration range of 10–20 w/v%. The authors could control the deposited strand dimensions by the hydrogel physical properties and operating parameters and achieved a mechanically stable scaffold with high cell viability (> 97%). The effect of printing speed on HA slurry with a solid volume fraction of 55% showed that the printing speed affected the shape and printing quality of the final scaffold [277]. A low print speed (3 mm/s) could not match the amount of extruding slurry and the printed lines piled up resulting in wider lines and smaller scaffold pore size than the actual model. On the other hand, fast printing speed (8 mm/s) caused the printed lines to be thinner or broken because of a higher stretching force at the same volume of extruding slurry. The best printing speed was found to be 5 mm/s for 55vol.% HA slurry. Another study on Al_2_O_3_ slurry also indicated that the viscosity, print speed, nozzle diameter and layer thickness are influential in printing quality and shape of the fabricated scaffolds [278]. Moreover, in temperature-dependent techniques, the temperature should be controlled not to affect the final quality of the objects. The selected temperature in the process depends on the types of materials such as melting temperature of metals and ceramics or glass transition of polymers. For example, the SLS-printed biphasic calcium phosphate (BCP) scaffolds may have wavy deformations in their structures due to the uneven temperature distribution involved in SLS process [279]. BCP is a mixture of HA and β-TCP, thus the HA decomposition and conversion of β-TCP to α-TCP can occur at the elevated temperatures produced during SLS printing.

Among the studies on process parameters, some have used systematical methodologies including DOE and response surface methodology (RSM) such as Taguchi, two-level factorial, Box–Behnken, and central composite designs [221, 222, 228, 230, 234, 244, 245, 247, 253–255, 260, 263, 266]. A response surface methodology is an optimization approach and a collection of statistical and mathematical approaches utilized to accomplish the multi-objective optimization procedure in various systems including materials, geometry and process.

## Conclusions

Nowadays, 3D printing has become a definite part of tissue engineering, due to its controllability on manufacture of designed porous structure and customizability. This is the result of integrating medical imaging, and computer modeling with fabrication systems. The steps usually taken in order to 3D print a bone scaffold include obtaining CT images of the bone with defect, providing 3D solid model of the defect, creating 3D model of bone scaffold internal structure by repeating unit cells in *x*, *y* and *z* directions, providing the scaffold external geometry through Boolean operation, converting the scaffold model to STL file format, slicing the model via computer algorithm, and 3D printing.

The challenges in manufacture of bone tissue scaffold via 3D printing are the bone defect geometrical complexity, the material properties, and the insertion of biomolecules and cells. Medical imaging and processing, and the resulting CAD model aid in overcoming the first challenge. However, regarding the printable materials, there is still limitation, particularly for bioprinting and when functionally graded materials are involved. There is a need for further improvement of 3D printing machines to make high strength and low modulus bone scaffolds. Furthermore, development of a software that is able to define the material property within the 3D solid model of a bone scaffold is also required. Furthermore, the optimization of internal architecture and material of a bone scaffold needs to be done because several variables exist both in the material design and geometry design. This will enable us to achieve superior performance, particularly when considering multiple objectives including mechanical strength, elastic modulus, permeability and bone growth. It is better to do the optimization, based on a given application which can be accomplished using CAD model of the scaffold and the bone with defect, along with finite element analysis via a parametric analysis. In this way, design of experiments can be employed to reduce the number of analyses and to aid in better interpretation.

There are several 3D printing technologies including laser-based, extrusion-based, and ink-based 3D printing, some of which can incorporate cells in the scaffold structure during the fabrication process. The 3D printing techniques involve a number of variables in their processing approaches which influences the characteristics of the fabricated bone scaffolds, hence there is a need for careful optimization of these factors with respect to the properties obtained. The most studied parameters are power of laser beams and scanning speed in laser-based approaches, particulate characteristics in powder-based techniques, printing speed and viscosity of ink or slurry in ink-based techniques or for manufacture of ceramic and composite scaffolds, and the nozzle dimensions in nozzle-based approaches. The process optimization is of particular importance when cells and biomolecules are involved in the manufacturing process. In the inkjet and extrusion-based bioprinting, the viscosity of the bio-ink, print speed, nozzle diameter and dispensing pressure are important factors that affect the mechanical stability of a scaffold and the fate of cells within the construct.

Looking into the future, even when the technology-related challenges are overcome, there will be a long distance from transforming research know-how into clinical products from which society can benefit. Therefore, the standardization of 3D-printed scaffolds needs to be accelerated.

## Data Availability

The data and material will be available on request.
